# Cretaceous-Tertiary Foraminifera and Palynomorphs from Djega Section and Inferred Paleodepositional Environments, Rio Del Rey Basin, Cameroon, West Africa

**DOI:** 10.1155/2018/6126528

**Published:** 2018-05-29

**Authors:** Njon Olivier Anoh, Mbanda L. Njoke, Ngoe D. Elangwe

**Affiliations:** ^1^Department of Geology, Mining and Environmental Science, University of Bamenda, P.O. Box 39 Bambili, North West Region, Cameroon; ^2^School of Economics and Management Science, North China Electric Power University, Huilongguan, Beijing, China; ^3^Institute of Geological and Mining Research, Yaounde, Cameroon

## Abstract

Late Cretaceous-Paleocene foraminiferans and palynomorphs were recovered from the upper section of the Djega outcrop in the Rio del Rey Basin. Only a few planktonic foraminiferan species of the genera* Heterohelix* and* Hedbergella* were recovered among an assemblage dominated by calcareous and agglutinated benthonics. Marine dinocysts are curiously absent from among the palynomorph assemblage, which consists dominantly of pollen grains from land plants (angiosperms and gymnosperms) and pteridophytic spores, together with a few fungal remains. Two benthonic foraminiferal assemblages that include the Campanian-Maastrichtian* Bolivina afra-Haplophragmoides talokensis* and the Paleocene* Anomalinoides umboniferus-Eponides pseudoelevatus* are well established at this outcrop. The palynomorphs include a few typical Late Cretaceous and typical Paleogene species, while the majority are long ranging forms that straddle the Cretaceous-Tertiary boundary. The foraminiferal and palynomorph biostratigraphic distributions permitted us to recognize the succession of Campanian-Maastrichtian and Paleocene strata and the Cretaceous-Tertiary boundary for the first time in this basin. Lithofacies change from a monotonous thick pile of shales below, succeeded by sandstones, frequently alternating with mudstone, above. This indicates a general fall in sea level during the Early Paleocene earlier reported within this subregion, and the boundary marks the start of the out building of the Niger Delta which the Tertiary Rio del Rey Basin is part of. Both microfossils and lithofacies analyses aided the reconstruction of an open marine, probably middle to inner neritic shallow and transitional intertidal paleodepositional environments for the sediments exposed at this outcrop.

## 1. Introduction

The Cretaceous-Tertiary (K/T) boundary, also referred to as the Cretaceous-Paleogene (K/P) or (K/Pg) boundary [1, 2], defines the end of the Mesozoic Era at the top of the Maastrichtian 65 million years ago. It is known that one of the largest mass extinction events of the Phanerozoic marked this boundary, and the record of this geologically historic event has been revealed at several stratigraphic sections over the world, most commonly from the subsurface and much less so from outcrop [1–3] due to obliteration by surface geologic processes (weathering and erosion). A large meteorite impacted the earth at the Yucatan Peninsula in Mexico and created the Chicxulub Crater in which the melted rocks have been dated 64.98 ± 0.05 Ma. The timing of this event coincides with the mass extinctions, reported from several regions of the world, and probably includes that which we are reporting here from the Djega outcrop in the Rio del Rey Basin, Cameroon. Unless not known to these authors, the Cretaceous-Tertiary boundary has not yet been established in this subregion from any outcrop section. However, several well-documented sections at which this boundary has been established from outcrop in other regions around the world can be referred [1, 2, 4–6], from Spain, Brazil, Haiti, Tunisia, Ghana, and many others.

As a petroliferous and integral part of the greater Tertiary Niger Delta, which is a world class hydrocarbon producer located within the Gulf of Guinea, the Rio del Rey Basin of Cameroon presents a stratigraphy that straddles the Cretaceous and Tertiary geologic time intervals. Hitherto thought to be an exclusively Tertiary basin adjoining the Niger Delta, a major significance of the present work is the description of Cretaceous sediments in the Rio del Rey Basin. This has set the stage for the amendment in the nearest future of the basin's stratigraphic framework. Moreover, locating the K/T boundary provides a rare opportunity for us to study an important global geologic event from this local section and to correlate it with others described elsewhere in the world. The use of foraminiferans and palynomorphs also significantly permitted the study of the trend of the biotic turnover event of these important microfossil groups at this geologic boundary.

The planktic foraminiferans are well known to be affected by even slight climatic fluctuations, and their fossilized calcareous shells are capable of holding a perfect record of such changes. They are therefore expected to have been severely affected by the impact of the large meteorite and any other global events that might have caused climatic changes and should serve as very good indices to study the biotic changes across this K/T boundary.

The initial objective of this work was to study the newly exposed upper section of the road at the Djega outcrop using foraminiferans and palynomorphs and compare the age with that of the lower strata previously dated by Njoh [7], Njoh and Petters [8], and Njoh et al. [9]. The Paleocene age was revealed at this newly exposed upper section. The objective therefore was extended to include a resampling and analysis of the section and establishing an approximate boundary between the last Cretaceous stratum and the first Paleocene stratum and so the K/Pg boundary at this outcrop.

## 2. Geologic Setting

**Figure 1:**
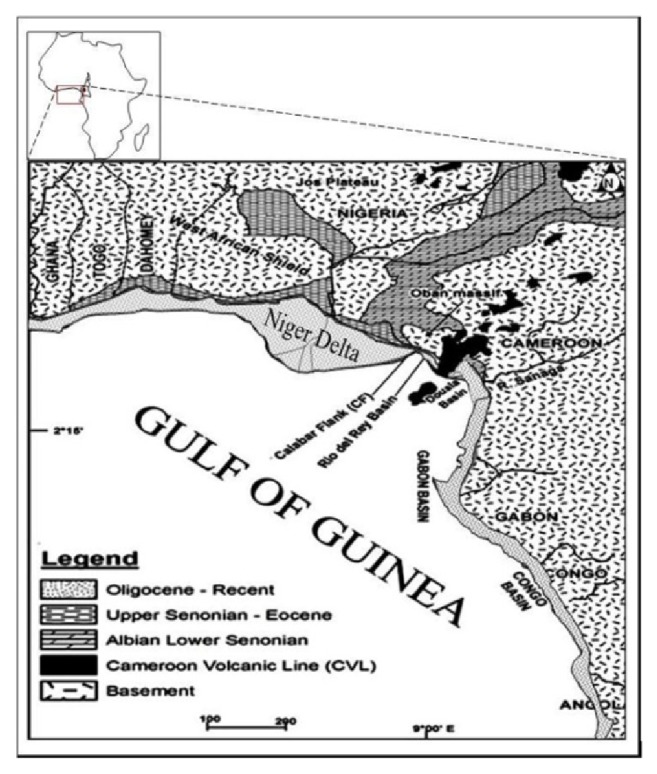
A regional map of the Gulf of Guinea showing the spatial relationship between the Rio del Rey and other coastal sedimentary basins within the subregion.

The Rio del Rey Basin, located at the extreme west coast of Cameroon, is one of the Equatorial Atlantic marginal basins within the Gulf of Guinea (Figure 1).

**Figure 2:**
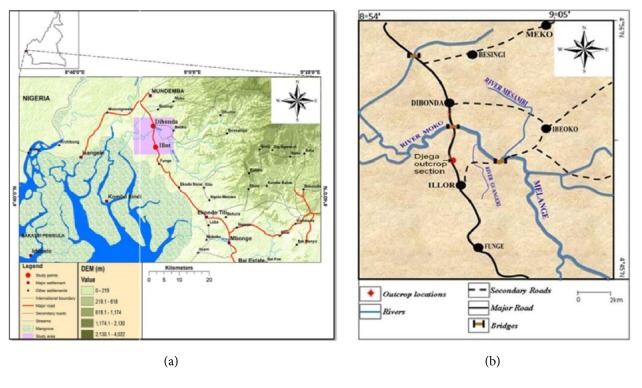
Map of the Rio del Rey Basin indicating the Illor-Dibonda area in the northern part of the basin.

**Figure 3:**
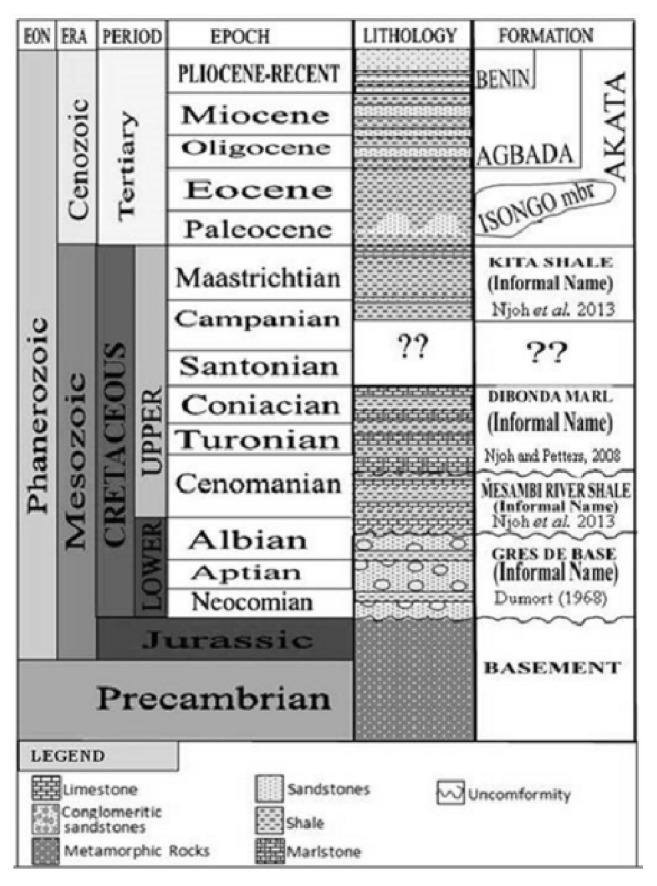
The lithostratigraphic framework of the Rio del Rey Basin with the Cretaceous section still under reconstruction.

It is genetically part of the Niger Delta and Calabar Flank Basins in the west and is bordered in the north by the Pre-Cambrian Oban Massif Basement and the Rumpi volcanics, in the south by the Rio Muni Basin in Equatorial Guinea and is separated in the east by the Cameroon Volcanic Line (CVL) from the Douala Basin. The Djega outcrop lies about 2 km northwest of Illor Village, along the Illor-Didonda road in the northern part of the Rio del Rey Basin (Figure 2). The tectonic and stratigraphic development of this basin is closely related to the rifting of Gondwana, the separation of South America from the African continent, the opening of the South Atlantic, and the formation of the Gulf of Guinea. It comprises the on- and offshore portions and a continental and marine sedimentary pile of over 6000 m thick. The deltaic sedimentary unit of Tertiary age in the Rio del Rey Basin is an eastward extension of the Niger Delta, while the Cretaceous is the continuation of the Calabar Flank. Much geologic information is available in the literature only from the Tertiary offshore segment of this basin because of the focus on hydrocarbon prospecting and production activities. Contrarily, very little is known of the Cretaceous stratigraphy of these sediments, which crops out along its northern flanks. However, Dumort [10] noted that the Neocomian-Albian basal conglomeratic unit of continental, fluviolacustrine sandstones, and conglomerates overlie the basement. Njoh et al. [11] dated an Albian-Cenomanian section using palynomorphs and suggested that the thick, dark-grey to black organic rich shales with a few thin limestone beds is overlying the basal conglomeratic unit. Earlier, Njoh and Petters [8] and Njoh et al. [9] had dated Upper Cretaceous sediments in this basin in which Turonian-Coniacian and Campanian-Maastrichtian sedimentary units were recognized. The Paleocene-Eocene to Recent Akata Formation is a prodeltaic, mainly marine shale unit overlying the Cretaceous and is overlain by the Oligocene-Miocene to Recent frequent alternations of sands and shales unit, the paralic Agbada Formation, which is the delta front. The Pliocene-Recent Benin Sandstone Formation is the uppermost unit of continental to coastal plain sandstones in this basin (Figure 3).

## 3. Materials and Methods

12 outcrop samples were selected at approximately 1 m intervals to represent the portion (18–30 m) of the outcrop and each sample was processed for both their foraminiferal and palynological content. 30 gms of each sample was processed for foraminiferans, and the indurated samples were first disaggregated and soaked with kerosene for up to 4 hours and in water with detergent overnight. The soaked samples were each washed over a 63 *μ*m sieve under a gentle stream of running water from a tap. The clean residues were collected and dried at 50°C in an oven. The dried samples were then sieved into the coarse, medium, and fine size portion to facilitate picking. Picking and identification were done under the binocular microscope with the aid of regularly used monographs from standard publications based on materials from the basins of the subregion and elsewhere [7, 12–15] amongst others. 20 gms of each sample was equally processed following standard palynological procedure as outlined in Traverse [16], Faegri and Iversen [17], and others, involving treatment with acids (HCl and HF) to remove carbonates and siliceous materials before sieving through a 10 *μ*m nylon mesh. The sieved samples were then oxidized with HNO_3_ before mounting on slides, which were then studied using the Leitz Diaplan microscope.

## 4. Results and Discussions

### 4.1. Lithofacies

**Figure 4:**
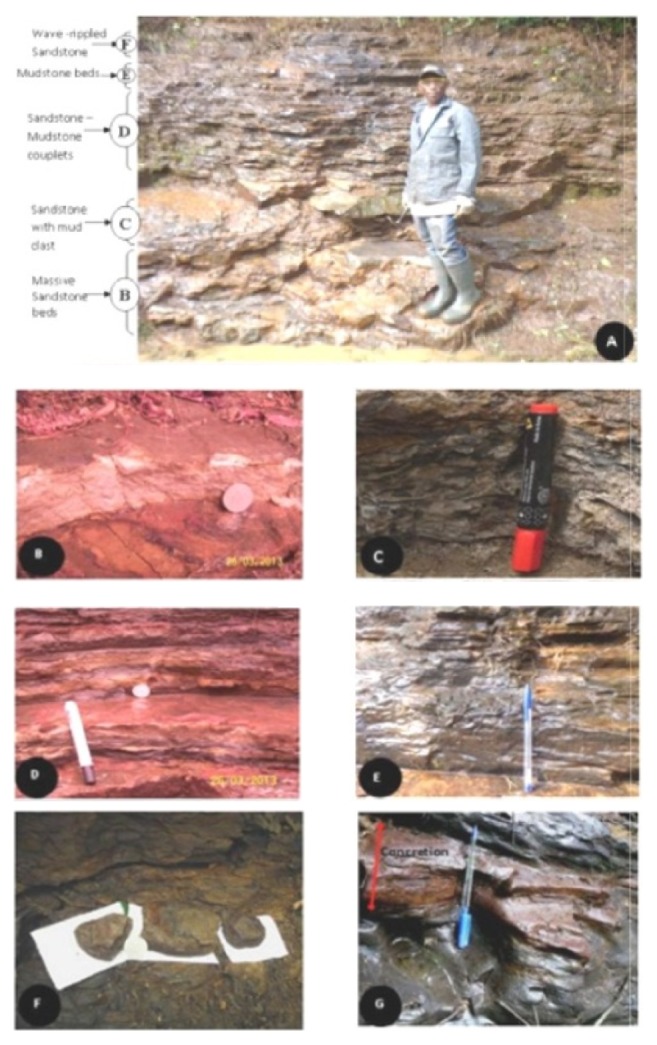
(A) The upper part of the Djega outcrop showing the frequently alternating beds of sandstones and mudstones. (B–F) show the various lithofacies of the upper part of the outcrop as indicated in (A). (G) A shale bed, iron stained, characteristic of the lower part of the Djega outcrop. (H) A bed of concretion, sometimes nodular frequently interbedding the shales.

The entire Djega outcrop section from base to top measures about 32 m thick; however, the present work has focused only on the upper section (18 m–30 m) of the outcrop (Figures 4(a)–4(g)). This section can best be considered as comprising a lower and an upper portion. Facing the River Moko and Dibonda direction, the lower portion on the right hand side descends from the road as a very steep slope that joins the River Djega valley below. The sediments here are exposed along small landslide escarpments, spring channels, and gullies that run down the slope. Lithologically, this portion is generally characterized by poorly bedded and indurated massive to thinly bedded shales. These shales become very fissile towards the top of this lower portion as one climbs out of the valley and onto the road. Several nodular and concretion beds (5 cm–7 cm) were also encountered at irregularly spaced intervals within this predominantly shale unit (Figures 4(f)-4(g)).

**Figure 5:**
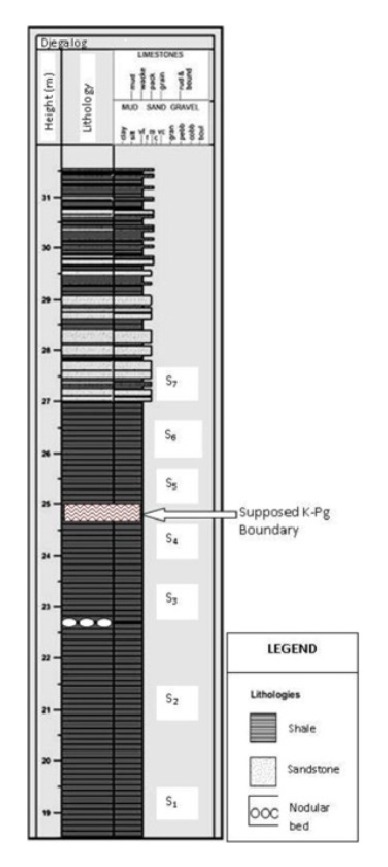
A composite lithologic log of the section of the Djega outcrop under study showing the position of the K/Pg, boundary.

The upper portion of this section includes the 4 m high roadside cut above (Figure 4(a)) which exposes a sequence of frequently alternating beds of sandstone and mudstone with thicknesses ranging between 1 and 51 cm and laminations of both lithologies. Each lithology is intercalated by very thin beds and laminations of the other three sandstone facies could be identified; (i) highly compacted, moderately sorted, generally clasts-supported medium grained sandstone beds; (ii) fine grained sandstone beds with frequent intercalations of black mud laminations described as sand-mudstone couplets [18]; and (iii) poorly sorted coarse-medium sandstone beds with inclusions of dark-grey fragments of mudstone described as mudclasts [19, 20] and sometime described as mottled (Figures 4(b)–4(e)). The surfaces of the sandstone beds are commonly characterized by symmetrical and asymmetrical ripples (Ripple index = 6). The shale generally dominates the upper part of this outcrop but occur as thin beds and laminations in the lower section which is equally dominated by the sandstones. Thin sandstone beds are also found within the shale and Slomka and Slomka [18] described them as mudstone-sandstone couplets (Figure 5). The entire outcrop describes a generally coarsening-upward sequence that begins with a thick pile of shale below (lower portion) and followed by a more sandstone predominated upper portion.

### 4.2. Microfossil Data

The preservation and species diversity of microfossils yield from the Djega outcrop is described as fair but the abundance is poor. Foraminifera, palynomorphs, ostracods, and echinoderm spicles including bivalves and their moulds were recorded from the fossiliferous intervals.

In this work, palynostratigraphy of the Djega outcrop was carried out for the first time as well as foraminifera biostratigraphy of the Tertiary section. Samples from the Cretaceous section were again analyzed for foraminifera and data compared with previous work. A total of 29 foraminifera taxa were recovered out of which 23 are benthics and only 6 planktics characterized by species of two strictly Cretaceous genera* Heterohelix* and* Hedbergella*. Of the 23 benthic foraminifera taxa 17 are Cretaceous (11, calcareous and 6 agglutinated) and 6 are tertiary (4 calcareous and 2 agglutinated). These samples also yielded a palynomorph assemblage of 33 taxa of which 17 are gymnospermous and angiospermous pollen grains, 10 are pteridophytic spores, and 4 are fungal spores and hyphae. Most of these spores and grains of pollen are derivatives of terrestrial plants but for a few swamp and salt marsh dwellers, the typical marine palynomorphs are noticeably absent. Some of these microfossils were identified down to species level, while others could only be identified to the genus. Amongst these, only a few short-ranging, age diagnostic taxa are included, some of which are well known to be typically Cretaceous and others typical Paleogene forms, while the rest are long ranging.

### 4.3. Biostratigraphic Analyses

The foraminifera and palynomorphs recovered from the Djega outcrop represent three categories: (i) restricted Late Cretaceous forms known not to have ranged any younger than the Maastrichtian, (ii) long ranging forms, commonly the palynomorphs that crossed the Cretaceous-Tertiary boundary, and (iii) those forms that have been recorded to have appeared for the first time only during the Paleocene and in younger strata.

#### 4.3.1. Foraminifera Biostratigraphy

The benthic foraminiferal assemblage obtained from the Djega outcrop and presented herein (Plate 1) have been recognized to undoubtedly belong to two assemblage zones of Petters [21], the Late Cretaceous (Campano-Maastrichtian)* Bolivina afra-Haplophragmoides talokensis* zone and the Early Paleocene (Danian)* Anomalinoides umboniferus-Eponides pseudoelevatus* zone. Typical members of the* Bolivina afra-Haplophragmoides talokensis* zone that have been recorded from the Djega outcrop include the several species of the genera* Preabulimina* and* Orthokastenia*,* Bolivina afra* (Petters) cf* Afrobolivina afra* (Reyment) and Kogbe and Me'Hes),* Bolivina explicata* (Cushman and Hedberg),* Neobulimina albertensis* (De Klasz, Magné and Rerat),* Nonionella robusta* (Plummer),* Valvulina umbilicatula *(D'Orbign),* Pseudonodosaria *sp. (Cushman and Renz),* Planulina nacatochensis *(Cushman),* Cibicides harperi *(Sandidge),* Textularia biafrae *(Reyment),* Trochamina texana *(Cushman and Waters),* Trochamina *sp.*, Haplophragmoides bauchensis *(Petters),* H. sahariensis *(Petters)*, H. numenhensi *(Petters), and* Ammobaculites coprolithiformis *(Shwager), while Cushman [12] and others had earlier used many of these forms to note the Upper Cretaceous sediments from several areas of the USA. Petters [21] remarked that the base of this zone was characterized by the first appearance of the species of* Preabulimina* and* Orthokastenia* in the lower Benue Trough and Dahomey Embayment in Nigeria and the top is marked by the complete disappearance of* Bolivina afra, Ammobaculites coprolithiformis, Cibicides harperi, Planulina nacatochensis, and *the genera* Preabulimna *and* Orthokastenia*. With their planktonic counterparts, this zone defines the Campanian-Maastrichtian age [22]. The species* Bolivina afra* cf* Afrobolivina afra* [14, 23] has been ascertained and used as a Late Cretaceous marker in the West African sedimentary basins.

**Figure 6:**
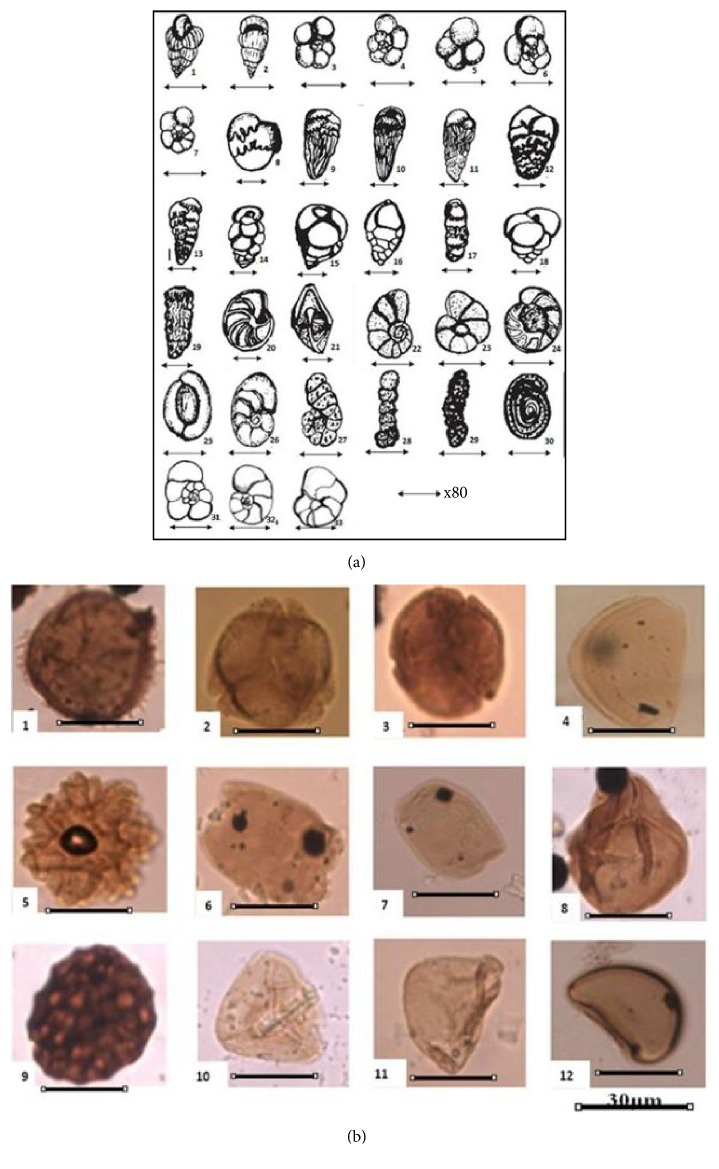
(a) Some common planktic and benthic foraminiferal forms recovered from the Djega outcrop represented by line drawings, not to scale: 1-2* Pseudotextularia elegans* (1 side view, 2 edge view), 3-4* Hedbergella planispira* (3 umbilical side, 4 spiral side), 5-6* H. monmouthensis* (5 umbilical side, 6 spiral side), 7* H. holmdelensis *(side view), 8–11* Bolivina afra* cf* Afrobolivina afra* (8 a megalospheric form, probably a juvenile, 9-10 side views, 11 apertural view), 12 and 13* Bolivina explicate *(side views), 14-15* Praebulimina robusta* (14 side view, 15 edge view), 16* Praebulimina laddi* (edge view), 17* Orthokarstenia ewaldi* (side view), 18* Praebulimina lata *(side view), 19 Orthokarstenia parva (side view), 20-21* Lenticulina secans* (20 side view, 21 edge view), 22-23* Planulina nacatochensis* (22 spiral side, 23 umbilical side), 24 Gavelinella guineana (spiral side), 25-26* Nonionella robusta* (25 spiral side, 26 umbilical side), 27* Ammobaculites subcretacea *(side view), 28-29* Ammobaculites coprolithiformis *(side views), 30* Haplophragmoides sp*. (umbilical side), 31* Epinoides *sp. (side view), and 32 and 33* Anomalinoides umboniferus*; (a) spiral view and (b) umbilical view. (b) Photo micrographs of some palynomorphs recovered from the Djega outcrop;** 1:*** Asplenium *sp.,** 2***: Syncolporites marginatus, ***3**:* Syncolporites marginatus, ***4**:* Longapertites marginatus, ***5**:* Ctenolophonidites costatus, ***6**:* Retidiporites magdeleinsis, *** 7**:* Retidiporites magdelensis, ***8**:* Acrostichum aureum, ***9***: Klukisporites pseudoreticulatus, ***10**:* Cyathidites *sp., **11***: Cyathidites *sp., **12:*** Laevigatosporites ovatus.*

The planktic foraminifera is characterized by two genera,* Heterohelix* and* Hedbergella,* amongst which there are* Heterohelix planata, H. globolusa, H. pulchra, Hedbergella holmdelensis, H. planispira,* and* H. monmouthensis;* others are* Pseudotextularia elegans* and* Rugoglobigerina *sp. (Figure 6(a)). The planktics are often more age diagnostic than their benthic counterparts but the short-ranging Maastrichtian marker species like* Rugoglobigerina macrocephala* did not feature in these samples. However, Njoh et al. (201a), Arz et al. (2004), and Petters [13] noted that members of the planktic foraminiferal assemblage above generally range from Turonian to the top of Maastrichtian. They have been used in the basins of the subregion to recognized Upper Cretaceous sediments and especially their association with the well-known age diagnostic benthic form,* Bolivina afra,* and others to diagnose the Campanian-Maastrichtian strata.

The benthic foraminifera were recorded from the upper section of the Djega outcrop and belong to the Early Paleocene (Danian)* Anomalinoides umboniferus-Eponides pseudoelevatus* Zone of Petters [21];* Anomalinoides umboniferus*,* Nonionella insecta, Textularia hockleyensis, Bulimina *sp., and* Pleurostemella paleocenica. *The base of this zone is said to coincide with the Cretaceous-Tertiary boundary and is marked by the extinction of almost all of the Maastrichtian species described above and the top also is marked by the disappearance of the Early Paleocene forms, notably* Anomalinoides umboniferus *and* Eponides pseudoelevatus* [21]. Njoh and Nkeme [24] and Njoh et al. (2009) included several of these species amongst others to describe Early Paleocene subsurface sediments from the neighboring Calabar Flank; meanwhile, the Paleocene Imo Shale and Kalambaina and Ewekoro Formations, respectively, were easily identified based on the occurrence of the members of this zone.

#### 4.3.2. Palynostratigraphy

We note here that although a few restricted Cretaceous and also Early Tertiary palynomorphs are included in this assemblage, a good number of the pollen and spores range across the Cretaceous-Tertiary, generally defining the Late Cretaceous-Early Tertiary period (Plate 2). Some Upper Cretaceous palynomorphs recovered from the Djega outcrop include* Retidiporites magdalensis *(Varma & Rawat,),* Longapertites marginatus *(Van Hoeken-Klinkenberg)*, Syncolporites marginatus *(Van der Hammen),* Ulmipollenites undulosus *(Wolf),* Monocolpopollenites spheroidite *(Pflug & Thomson)*, Ctenolophoridites costatus *(Van Hoeken-Klinkenberg)*, Asplenium *sp.*, Cyathidites *sp.*, Deltoidospora *sp.*, Laevigatosporites ovatus *(Ibrahim)*, Filtrotrilites *sp., and* Psilatriporites *sp., among others (Figure 6(b)). Lawal and Moullade [25], Ojo and Akande [26, 27], and Onuduku and Okonsu [28] independently used a similar palynomorphs assemblage to recognize the Early and Late Maastrichtian sections in the upper Benue Trough of Nigeria. Salami [29], Edet and Nyong [30], Atta-peters and Salami [31, 32], and Atta-Peters et al. [33], respectively, assigned the Maastrichtian age the Lower Coal Measures in the Anambra Basin and to the Nkporo Formation of the Calabar Flank of Nigeria and sections in the Tano Basin, western Ghana, based on the recovery of similar palynomorphs from these localities. Meanwhile, Hengreen [34] had earlier used a similar species assemblage to establish an Upper Cretaceous microfloral relationship between Africa and South America.

The* Longapertites marginatus, Syncolporites marginatus, Retidiporites magdalensis, Monocolpopollenites spheroidite, Ulmipollenites undulosus,* and* Asplenium *sp. species which are well known to occur in the Upper Maastrichtian have equally been reported from Paleocene and younger strata.* Syncolporites marginatus* is a very important element of the assemblage Mbesse [35] used to establish the Paleocene age to sedimentary section of the Nkapa Formation of the Douala Basin.* Deltoidospora *sp. and* Verrucatosporites *sp. occur among the Paleocene species recovered from offshore Abidjan, Cote d'Ivoire, while several species of* Longapertites, Retistephanocolpites,* and* Acrostichum* characterize the Late Paleocene Patala and Lakhra Formations, respectively, in northern Pakistan [36].

### 4.4. The K/T Boundary at Djega Outcrop

It is clear from the preceding analysis that the microfossils recorded from this outcrop penetrated and have established a close and sequential occurrence of the Upper Cretaceous (Campanian-Maastrichtian) and Paleocene (Danian) sedimentary strata and that in between them lies the K/T boundary. The logged section of the outcrop and the vertical distribution of the microfossils point out that this boundary lies between sample S4 at about 24 m of height and sample S5 at about 25 m (Figure 5). The foraminifera have provided a better refined position of the boundary on this outcrop. Sample S4 saw the abrupt disappearance (extinction) of all the Cretaceous planktics, especially the species of* Heterohelix* and* Hedbergella* and many benthics that typify the Campanian-Maastrichtian age in this subregion and elsewhere and are known not to have ranged any younger than the Maastrichtian. Some of these benthics include* Bolivina afra, Bolivina explicata, Orthokastenia clavata, Orthokastenia ewaldi, Preabulimina laddi, Preabulimina lata, and Textularia biafrae*. This mass extinction was immediately followed above in sample S5 by the first appearances of typical Paleocene taxa:* Anomalinoides umboniferus*,* Pleurostomella paleocenica, and Nonionella insecta. *The Cretaceous planktics and benthic foraminifera were lastly recovered at this outcrop from sample S4 but were completely absent or had disappeared from Sample S5 1 m immediately above. Sample S5 equally witnessed the appearance for the first time of the Paleocene exclusively benthic forms indicating that at this outcrop there is rather an abrupt mass extinction of the Cretaceous foraminifera (Figure 6(a)). The step-by-steep trend in the disappearances of Cretaceous forms before the K/T boundary as reported from elsewhere cannot be supported or simply be disregarded in this work.

The palynomorphs on the other hand have equally confirmed the penetrative Paleocene-Upper Cretaceous ages at this outcrop by the record of the forms named above known to have straddled the K/T boundary and cooccur below with typical Upper Cretaceous forms and above with typical Lower Tertiary forms. The specimens recorded from this outcrop that define the Cretaceous are* Cycadophytes* sp.*, Filtrotriletes nigeriensis, *and* Ctenolophoridites costatus*.

These species occurred only up to sample S3 and not beyond; meanwhile, the typical Lower Tertiary forms,* Zonocostites minor* and* Klukisporites pseudorecticulatus*, had their first appearance in sample S5 and probably* Asplenium *sp. which was encountered only in sample S7 (Figure 6(b)).

### 4.5. Paleodepositional Environment

An integrated lithofacies and microfossil data analysis has permitted us to diagnose the environments into which the sediments exposed at the Djega outcrop were deposited.

Foraminifera is an exclusive group of marine microorganisms and the presence their fossil forms in the sediments studied in this work is a simple but direct indication that they are of a marine origin. A comparative synoptic analysis of the planktics/benthics and the agglutinated/calcareous benthics ratios, respectively, generally show that the sediments were deposited in an environment that ranges from an open marine that is not too deep, probably middle neritic, to a shallow near-shore inner neritic. Njoh and Agbor [37] recovered fossilized fish and turtle-like heads in the massive shale beds of the lower portion described at this outcrop which were used to infer a deep water environment (Middle neritic) in which these ancient mega organisms swamp. The palynomorphs recovered from these sediments are mainly derivatives of swamps and land dueling plants, indicating the proximity of the paleo-depositional environments to the continent.

Two main lithologic types, sandstone and shale, have been described above as characterizing the sedimentary deposits exposed at the Djega section. The thinly bedded black and very fissile shales and massively bedded dark to dark-grey shales that characterized the lower portion of the outcrop are inferred to have been deposited in a relatively deep water environment. The upper portion of the outcrop comprises a frequent alternation between sandstones and shales in which some of the sandstones are commonly rippled, sandstone-mudstones couplets and mudclasts are common and this indicates a near-shore transitional environment of deposit. Detailed lithofacies descriptions and inferred depositional environments were carried and summarized on Table 1 as modified from Njoh and Agbor [37].

From above, the sediments studied at the Djega outcrop are inferred to have been deposited in a varied environment which ranged from a shallow near-shore (neritic) transitional environment characterized by lagoons, foreshore, and intertidal deposits for the upper section to a deeper open marine probably middle marine for the lower portion of this outcrop. The entire outcrop therefore represents a coarsening-upward sequence depicting a fall in sea level which also marked the onset of the outbuilding of the tertiary delta.

## 5. Summary and Conclusion

The portion of the Niger Delta Basin of Nigeria that extends eastward into the territory of Cameroon is known as the Rio del Rey Basin [38]. While the Niger Delta proper is exclusively Tertiary in age, the Rio del Rey Basin slightly differs as it occurs both off- and onshore and ranges from Cretaceous to Tertiary in age. Both basins are very important hydrocarbon producers and until present, they are producing exclusively from their Tertiary portions wherein the sedimentary strata consequently have been well studied.

We highlight in this work the Cretaceous sediments of the Rio del Rey Basin which outcrop onshore, contrarily to most previous publications that portray the basin as exclusively tertiary and restricted offshore. The tertiary sediments of this basin have previously been described uniquely from offshore, but this work has revealed that Paleocene strata are also exposed onshore. Apart from demonstrating the level at the Djega outcrop section of the Rio del Rey Basin where the Upper Cretaceous strata are overlain by the Paleocene, the aforementioned highlights will clearly disperse some misleading concepts erroneously propagated in past literature.

Foraminifera and the palynomorphs recovered from the studied outcrop have helped us to date Lower Tertiary (Paleocene) and the Upper Cretaceous (Campanian-Maastrichtian) sediments in the Rio del Rey Basin. These microfossils in concert have equally permitted the tracing of an approximated contact between these two important units and therefore the K/Pg boundary in this region from outcrop. We note that many foraminifera species in this basin never crossed the boundary unlike the palynomorphs in which many did cross. This indicates that the fauna turnover event at this time was much more noticeable in the foraminifera than with the palynomorphs. Apart from the frequent reports on petroleum geology, the K/Pg boundary and events have permitted us to report yet another yet global aspect of the geology of this basin and correlate it with others, especially outcrops that have been studied in Brazil, Mexico, Spain, Ghana, and other parts of the world.

Probably due to field conditions which did not permit a more refined sampling, we conclude that no sharp contact or physical boundary was encountered on the field to directly point the K/T boundary at a particular bedding plane or surface of unconformity as in similar studies elsewhere [1, 2] but our microfossil interpretations have located this boundary within a narrow interval of 2 m between samples S4 at 22 m and S6 at 24 m on the Djega outcrop in the Rio del Rey Basin southwestern Cameroon.

Analysis of the lithofacies combined with the microfossil data suggests that these sediments were deposited in an open shallow marine (middle to inner neritic) and transitional intertidal environments.

## Figures and Tables

**Table 1:** Summary of the lithostratigraphic description and inferred depositional environment for sediments studied.

Facies code	Facies name	Lithology	Structure/fossil/mineralogy	Depositional setting/Environment
Sm	Massive sandstones	Fine-medium moderately to well sorted quartz arenite with sub angular to sub-rounded grains	Massive, over 90% quartz, >5% feldspars, micas and rock fragments. Glauconite is present	Shoreface setting/Shallow marine environment

Sw	Wave rippled sandstone	Fine-medium moderately well sorted sandstones with sub angular to sub-rounded grains	over 90% quartz, >5% feldspars, micas and rock fragments. Glauconite and wave ripple marks present	Flood-tidal to ebb-tidal deposits/Lagoonal to shallow marine environment

Smc	Massive Sandstone with mudclast	Fine-medium moderately to well sorted sandstones with sub angular to sub-rounded grains, hosting grey to black millimetric mudstone fragments	Massive, with sharp bed boundaries	Flood-tidal to ebb-tidal deposits/Lagoonal to shallow marine environment

SM	Sandstone-Mudstone couplets	Sandstone with intercalations of thin laminations of dark grey to black mudstones	Parallel laminations with sharp bed boundaries	Wash-over, Flood-tidal to ebb-tidal deposits/Lagoonal to shallow marine deposits

Fm	Massively bedded shale	Dark to dark-grey with iron stained colorations. Nodular and concretion beds at irregular intervals	Poorly bedded with visible tiny pyrite crystals. Fish head and turtle-like fossils	Deep Marine environment

Fl	Parallel thin-laminated shale	Dark grey to black highly fissile shale. Forms intervals that range in thickness from 20 cm to 2.5 m	Parallel laminations/bedding, contains tiny pyrite crystals. Fish head and turtle-like fossils, as well as bivalve moulds present	Deep Marine environment
